# Draft genome sequence of the epiphytic *Bacillus pumilus* strain App11D isolated from the surface of *Alhagi pseudoalhagi* (Bieb.) Fisch. seeds

**DOI:** 10.1128/mra.00529-25

**Published:** 2025-10-14

**Authors:** Vladimir K. Chebotar, Ivan A. Lunegov, Maria S. Gancheva, Dmitriy V. Kudryavtcev, Olga A. Bortsova, Elena P. Chizhevskaya, Veronika N. Pishchik

**Affiliations:** 1 All-Russia Research Institute for Agricultural Microbiology, St. Petersburg, Russia; 2 Peter the Great St. Petersburg Polytechnic University, St. Petersburg, Russia; 3 Saint Petersburg State University, St. Petersburg, Russia; University of Strathclyde, Glasgow, United Kingdom

**Keywords:** draft genome, epiphytic, *Bacillus pumilus*, App11D, *Alhagi pseudoalhagi*, seeds

## Abstract

The genome sequence of the epiphytic bacterium *Bacillus pumilus* App11D, isolated from the seeds of *Alhagi pseudoalhagi*, has been obtained. The sequencing was performed using Illumina technology, and the genome was assembled *de novo* with SPAdes software.

## ANNOUNCEMENT

The epiphytic strain App11D was isolated from the seed surface of the drought-tolerant plant *Alhagi pseudoalhagi* (Bieb.) Fisch., collected in the Neftekumsky district of Stavropol Territory (near the village of Achikulak, N 44°33′43″, E 44°49′59″), following the protocol described in (1). 10 g of seeds were sonicated (Bandelin, 50 Hz, 10 min) in 100 mL sterile water. Serial dilutions (0.1 mL) were plated on LB and Ashby media, followed by 3–5 days of incubation at 28°C. Genomic DNA extraction was carried out using the Wizard Genomic DNA Purification kit (Promega, USA) according to the manufacturer’s instructions. Library preparation was performed with 100 ng of extracted DNA using the KAPA HyperPlus kit (Roche, Switzerland). Final purification was conducted with KAPA Hyperpure beads (Roche, Switzerland). The library size distribution and quality assessment were evaluated with the High Sensitivity DNA kit (Agilent Technologies, USA), while the Quant-iT DNA Assay kit (Thermo Fisher Scientific, USA) was used for quantification. Sequencing was performed on the NextSeq 1000 platform (Illumina, USA) using NextSeq 1000/2000 P1 Reagents (300 cycles) with 2% PhiX spike-in control, generating 1,139,301 read pairs (150 bp length).

The quality of the raw reads was assessed using FastQC v0.11.9 (2), which indicated high-quality data with no need for additional trimming or filtering. Genome assembly was performed using SPAdes v3.14.1 (3), yielding 59 contigs with an N50 of 1,836,326 bp. All Bacillus reference genomes were downloaded from NCBI RefSeq (4), and FastANI v1.34 (5) was used to perform whole-genome comparisons between these genomes and our assembly. The App11D strain showed the highest similarity to *Bacillus pumilus* SAFR-032 (GCF_000017885.4) with an average nucleotide identity of 95.5%. Scaffolding was performed by aligning contigs to the reference genome with Ragtag v2.1.0 (6), followed by the removal of scaffolds shorter than 500 bp. Plasmid detection was carried out on the resulting scaffolds using PlasmidFinder-2.0 (7) and PlasFlow-1.1 (8), but no plasmids were identified. Assembly quality metrics were evaluated with QUAST v5.1.0 (9), the samtools coverage tool v1.21 (10), and BUSCO v5.2.2 (bacillales_odb10) (11). Genome coverage was calculated by mapping reads to the assembly with minimap2 v2.28 (12). The draft genome assembly achieved 94.3× coverage with only 5 scaffolds totaling 3,616,178 bp (N50 value = 3,610,638 bp). The GC content was calculated at 42.05%, and BUSCO analysis confirmed 100% completeness of the assembly. Unless otherwise specified, default software parameters were applied.

The genome annotation was carried out with the NCBI Prokaryotic Genome Annotation Pipeline v6.9 (13), resulting in the identification of 3,705 genes. Gene clusters associated with secondary metabolite biosynthesis were analyzed using antiSMASH v7.1.0 (14) with all additional features enabled. The analysis identified gene clusters predicted to encode biosynthetic pathways for lichenysin, zwittermicin A, schizokinen, fengycin, bacilysin, and bacillibactin production. Previous studies have demonstrated the potential of *B. pumilus* strains to promote plant growth by producing phytohormones and other bioactive compounds that improve plant biometric traits and stress tolerance (15). Given its isolation from the drought-resistant *A. pseudoalhagi*, strain App11D, represents a promising candidate for investigating plant growth-promotion and stress-alleviation properties.

## Data Availability

The genome sequence and raw reads were deposited in NCBI and can be accessed under BioProject PRJNA1194473 (SRA SRR31618171, assembly GCA_048595385.1).
